# The Pattern Dynamics of Propagation Models in Complex Networks

**DOI:** 10.3390/e28040370

**Published:** 2026-03-25

**Authors:** Xuerui Zhu, Xinlin Chen, Le He, Linhe Zhu

**Affiliations:** 1 School of Physics and Electronic Engineering, Jiangsu University, Zhenjiang 212013, China; 2 School of Mathematical Sciences, Jiangsu University, Zhenjiang 212013, China; 3 School of Mathematics, Shandong University, Jinan 250100, China

**Keywords:** rumor propagation, Turing pattern, homogeneous network, heterogeneous network

## Abstract

Based on the detrimental impact of network rumors, we employ the Laplacian matrix to reconstruct a diffusion term and establish a rumor propagation model that extends from traditional physical space to cyberspace. In this paper, we analyze the occurrence conditions for Turing instability in both homogeneous and heterogeneous networks and generalize the necessary conditions for Turing instability in higher-order systems. Through numerical simulations, we demonstrate the existence of Turing patterns. Additionally, we explore the intricate nature of Turing patterns within various network structures. The findings from the homogeneous network indicate that the BA scale-free network is better suited for real-world scenarios. When implementing rumor-refuting policies, prioritizing individuals in key nodes can effectively control rumors throughout the entire network, significantly enhancing rumor control efficiency. The results on heterogeneous networks demonstrate that altering the key layer instead of modifying the entire three-layer network structure can also achieve the goal of rumor governance. This discovery substantially reduces the complexity associated with rumor control.

## 1. Introduction

The dissemination of rumors not only jeopardizes personal property safety but also undermines social stability. During the COVID-19 (*Corona*-*Virus*-*Disease* 2019) pandemic, in addition to the spread of the disease, some individuals blindly embraced the rumors that smoking and drinking can effectively prevent viral infection, leading to excessive indulgence in these activities, which ultimately weaken immune systems and increase susceptibility to infection, thereby affecting the transmission process of infectious diseases. In the face of the uncontrollable situation of pathogens, many scholars have proposed strategies for early identification and timely optimization of control [1,2,3,4], but these did not take into account the change in people’s attitudes during the disease transmission process, and they ignored the influence of information on actual behavior. Therefore, studying the dynamics of rumor propagation and safeguarding public opinion is a crucial pillar for upholding societal well-being and national stability.

The widespread reaction–diffusion phenomenon in nature provides us with important theoretical inspiration for exploring the mechanism of rumor dissemination. The reaction–diffusion equation, as a mathematical model describing the spatial and temporal distribution changes resulting from the diffusion of substances, has been applied in numerous scientific fields since its inception. In addition, the process of rumor dissemination shares profound similarities with the reaction–diffusion phenomenon in nature. The spread of rumors essentially revolves around two aspects: “reaction” and “diffusion”, namely individual contact and information flow. Therefore, more and more scholars are analyzing the evolution mechanism of rumors in the temporal and spatial dimensions by establishing reaction–diffusion models. Huo constructed a dual-media rumor dissemination model and provided a strict analytical framework for the global stability of the system [5]. Zan constructed a dual-rumor dissemination model, which more comprehensively analyzed the overall dissemination state of rumors and explored rumor control based on network topology [6]. Qiu incorporated the influence mechanism among users in a social network into the traditional compartment model [7]. By examining the impact of parameter variations during the study of rumor propagation, a method for controlling rumors was devised based on this theoretical framework. Turing patterns refer to the patterns that can be described by a reaction–diffusion system, initially proposed by the mathematician Turing [8]. If the system undergoes Turing bifurcation, altering the critical control parameters will result in the disruption of spatial symmetry, which is an essential condition for generating Turing patterns. Currently, Turing patterns have been extensively studied in various fields such as material science [9], ecological environment [10], and disease transmission [11]. Especially in the field of population modeling, predator–prey systems have attracted many researchers to conduct investigations. They incorporate multiple complex factors such as Allee effect [12,13], cross-diffusion [14,15], and time-delay effect [16,17] to further explore their influence on the mechanism of pattern formation. In terms of rumor propagation, studying Turing patterns allows for visualizing the distribution structure of different types of individuals within space [18], providing crucial decision-making foundations and theoretical guidance for managing rumors. By analyzing the conditions for Turing instability, it was concluded that periodic diffusion behavior influences the distribution of crowd gathering areas during rumor propagation. Li [19] incorporated media correction and self-correction factors into her model on rumor propagation. By deriving amplitude equations [20,21,22], scholars have revealed that specific parameters control the shape of Turing patterns and have analyzed how changes in cross diffusion coefficient affect pattern structures. It was suggested that increasing cross diffusion accelerates rumor spread.

The traditional model of rumor propagation considers the continuous space as the propagation background, while the current mainstream method of rumor propagation is through network platforms. With the increasing number of new users on the platforms, the harm caused by network rumors has exponentially increased. Currently, based on scholars’ different priorities, we have divided their research into two main directions. On one hand, some scholars analyzed the spread patterns of rumors by mining specific characteristics within groups. The work from Xiang [23] introduced game theory to predict rumor popularity and clarify the underlying mechanisms behind rumor propagation in dynamic network interactions. On the other hand, other scholars leveraged the high similarity between rumor propagation and infectious diseases, considering individuals as nodes in a network and establishing a transmission dynamics framework for modeling network rumor propagation. The researchers extended the single-layer network to double-layer and multi-layer networks to explain the process of rumor dissemination and counter-rumor information [24]. In addition, regarding the transmission mechanism, scholars incorporated more realistic assumptions such as hesitation psychology [25] and time-delay effects [26], thereby enhancing the application value of the model. In order to study both online and offline networks’ spread of rumors and anti-rumors, Du [24] took social reinforcement and interest decay effects into account. Through Monte Carlo simulation, it was concluded that rumor propagation speed was influenced by network structure with corresponding rankings provided. To break the assumption about relatively simple mechanisms between the propagation of different nodes, Chen [25] combined refutation methods with the process of rumor spreading and expanded the population classifications for rumor propagation. The results demonstrated varying restraining effects on network rumor spread depending on different refutation methods.

In previous studies on rumor propagation, the majority of scholars focused their efforts on constructing a conventional reaction–diffusion system to investigate the characteristics of online rumor propagation. However, there has been relatively limited research conducted on network Turing patterns of rumor spatiotemporal propagation. This paper aims to examine the impact of diverse network structures on the formation of Turing patterns and generalize the necessary conditions for Turing instability to occur in high-dimensional systems.

The structure of this paper is outlined as follows. In Section 2, we introduce the reasons for the evolution of the model in detail, focusing on the establishment process of the rumor propagation model on the network. In Section 3, we give the existence condition of the positive equilibrium point combined with the figures. In Section 4, we analyze the existence conditions of Turing instability in homogeneous and heterogeneous networks. In Section 5, we discuss various properties of Turing patterns through numerical simulation, which enhanced the efficiency of rumor propagation control. Section 6 evaluates the practical applicability of the proposed model using a dataset of COVID-19-related tweets from Indonesia, while Section 7 presents the final conclusions of this research.

## 2. Model Formulation

The rumor propagation model focuses on studying the interaction and influence among individuals within a population, providing a theoretical foundation for predicting development trends and assessing the effectiveness of control measures. The dissemination of rumors shares fundamental structural similarities with the spread of infectious diseases like COVID-19, as both phenomena are driven by interpersonal contact and subsequent state transitions within a population. Drawing on the classic SIR (Susceptible–Infected–Recovered) framework pioneered by Daley and Kendall [27], who established the theoretical bridge between epidemic modeling and information diffusion, we categorize the population into three distinct groups: S(t) represents the susceptible individuals who are targeted by rumor propagation; I(t) represents the spreading individuals who initiate and spread rumors; R(t) represents the immune individuals who possess knowledge of truth or have lost interest in rumors.

By mapping these epidemiological states to rumor propagation, we can intuitively describe how a rumor “infects” a susceptible population through contact with spreaders [18]. Initially, a homogeneous model that disregards spatial behavior is established:

(1) $$\left\{\begin{array}{c} \frac{dS(t)}{dt}=rS\left(t\right)\left(1-\frac{S(t)}{K}\right)-\beta S\left(t\right)I^{2}\left(t\right)-\mu_{1}S\left(t\right),\\ \frac{dI(t)}{dt}=\beta S\left(t\right)I^{2}\left(t\right)-\mu_{2}I\left(t\right)-\alpha I\left(t\right),\\ \frac{dR(t)}{dt}=\alpha I\left(t\right)-\mu_{3}R\left(t\right),\\ S\left(0\right)>0,I\left(0\right)>0,R\left(0\right)>0. \end{array}\right.$$

There exists the following two salient points in the model setup.

• $rS\left(1-\frac{S}{K}\right)$ represents the Logical growth term. The increase in network users can not be infinite, subject to the limitations of science and technology. To better describe the growth process, it is necessary to introduce a logical equation that considers both internal growth rate and carrying capacity.

• $\beta SI^{2}$ represents the nonlinear propagation factor. Under the influence of the social reinforcement effect [28], the spread of rumors in social networks does not occur through a single contact but rather through repeated interactions. Due to individuals’ skeptical attitude towards new information, they do not immediately believe the rumors. Instead, they will only believe and adopt the information upon their next contact, which in the dynamics of infectious diseases is explained as “non-linear incidence” [29,30].

Next, we will specify the meaning of each model parameter through the following Table 1. Every parameter is positive.

The current primary mode of rumor propagation is through cyberspace. However, the conventional ordinary differential equation system (1) fails to capture the spatial influence on rumor propagation. Therefore, we propose the following reaction–diffusion system based on continuous space, and the Neumann boundary conditions with 0 flux are used [31]:

(2) $$\left\{\begin{array}{cc} \frac{\partial S\left(t,x\right)}{\partial t}=d_{1}\Delta S+rS\left(t,x\right)\left(1-\frac{S\left(t,x\right)}{K}\right)-\beta S\left(t,x\right)I^{2}\left(t,x\right)-\mu_{1}S\left(t,x\right),\; & t>0,x\in \Omega ,\\ \frac{\partial I\left(t,x\right)}{\partial t}=d_{2}\Delta I+\beta S\left(t,x\right)I^{2}\left(t,x\right)-\mu_{2}I\left(t,x\right)-\alpha I\left(t,x\right),\;\;\;\; & t>0,x\in \Omega ,\\ \frac{\partial R\left(t,x\right)}{\partial t}=d_{3}\Delta R+\alpha I\left(t,x\right)-\mu_{3}R\left(t,x\right),\;\;\;\;\;\;\; & t>0,x\in \Omega ,\\ \frac{\partial S(t,x)}{\partial v}=\frac{\partial I(t,x)}{\partial v}=\frac{\partial R(t,x)}{\partial v}=0,\;\;\;\;\;\; & t>0,x\in \partial \Omega ,\\ S\left(0,x\right)>0,I\left(0,x\right)⩾,≢0,R\left(0,x\right)⩾0,\;\;\;\;\;\;\;\; & x\in \Omega , \end{array}\right.$$

where $d_{1},d_{2}$ and $d_{3}$ represent the diffusion coefficients of S,I and *R*, respectively. The diffusion term is expressed as the product of the Laplacian operator $\Delta S=\frac{\partial^{2}S}{\partial x^{2}},$ $\Delta I=\frac{\partial^{2}I}{\partial x^{2}}$ and $\Delta R=\frac{\partial^{2}R}{\partial x^{2}}$ multiplied by their corresponding diffusion coefficients for different classes of individuals. Ω denotes a bounded region with a smooth boundary expressed as ∂Ω.

In fact, it is important to note that rumor propagation is modeled using reaction–diffuision equations, which can only explain phenomena in continuous space. In the field of biomathematics, a reaction–diffusion system is established to analyze problems through the spatial migration of populations, requiring actual contact between individuals in physical space. For instance, the predator–prey model necessitates contact between prey and predator to discuss population survival and extinction, while the infectious disease model requires contact between susceptible and infected individuals for disease spread. Historically, rumors spread through word-of-mouth aligning with the requirements of a reaction–diffusion system to explain population flow behavior in continuous space. However, in today’s Internet era where rumors primarily spread through social networks, the application of reaction–diffusion equations becomes inappropriate within this context. Therefore, we consider the discrete continuous space as the network structure with internal homogeneity of nodes. That is to say, the Laplacian operator is discretized to obtain the Laplacian matrix $L={\left(l_{ij}\right)}_{N\times N}$, which serves as a representation of the diffusion environment. The following system is constructed based on the homogeneous network structure [19]:

(3) $$\left\{\begin{array}{cc} \frac{dS_{i}\left(t\right)}{dt}=d_{1}\sum_{j=1}^{N}l_{ij}S_{j}\left(t\right)+rS_{i}\left(t\right)\left(1-\frac{S_{i}\left(t\right)}{K}\right)-\beta S_{i}\left(t\right)I_{i}^{2}\left(t\right)-\mu_{1}S_{i}\left(t\right),\; & i\in \hat{\Omega },\\ \frac{dI_{i}\left(t\right)}{dt}=d_{2}\sum_{j=1}^{N}l_{ij}I_{j}\left(t\right)+\beta S_{i}\left(t\right)I_{i}^{2}\left(t\right)-\mu_{2}I_{i}\left(t\right)-\alpha I_{i}\left(t\right),\;\;\; & i\in \hat{\Omega },\\ \frac{dR_{i}\left(t\right)}{dt}=d_{3}\sum_{j=1}^{N}l_{ij}R_{j}\left(t\right)+\alpha I_{i}\left(t\right)-\mu_{3}R_{i}\left(t\right),\;\;\;\; & i\in \hat{\Omega },\\ S_{i}\left(0\right)>0,I_{i}\left(0\right)⩾,≢0,R_{i}\left(0\right)⩾0, & i\in \hat{\Omega }, \end{array}\right.$$

where the subscript set of network nodes is denoted by $\hat{\Omega }$. In previous studies of network information propagation, the Turing pattern has been investigated by the aforementioned homogeneous network for modeling, where there existed only one type of connection relationship between different network nodes. This implies that different populations share the same diffusion direction, often resulting in challenges when attempting to discern population relationships within the system. In contrast, heterogeneous networks enable integration of multiple types of population relationships, thereby introducing variability into the diffusion direction among different populations.

**Figure 1:**
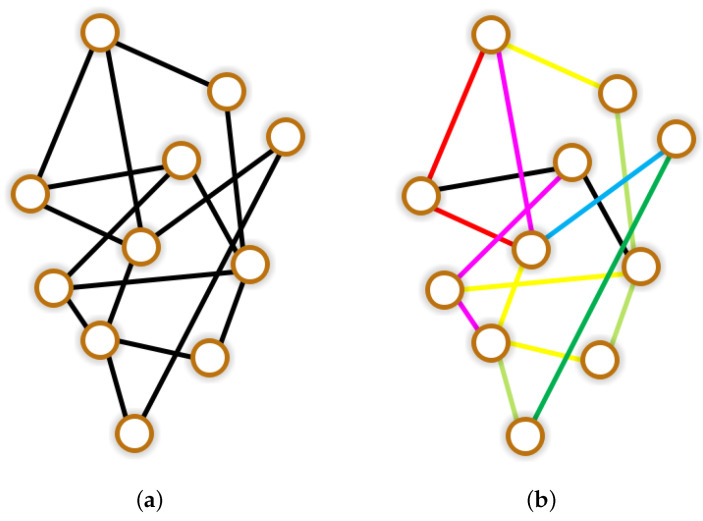
Homogeneous and heterogeneous network structures.

Figure 1a depicts the homogeneous network, where black edges connect the nodes. Reactions take place within the nodes, while diffusion occurs along the edges. Figure 1b shows the heterogeneous network with edges of different colors replacing some of the black ones. The red connection line indicates exclusive flow for *S*, while the green and blue lines signify exclusive flow for *I* and *R*, respectively. Therefore, by considering the superposition of colors, we give the meaning of the edges in different colors.

- Yellow: Only *S* and *I* are allowed for flowing.
- Magenta: Only *S* and *R* are allowed for flowing.
- Cyan: Only *I* and *R* are allowed for flowing.

In this case, the permissible diffusion of individuals for each connected edge is different, which means that different connected edges are not homogeneous, highlighting the heterogeneity among different connected edges. At this time, we replace the original homogeneous with the heterogeneous network structure [32,33]. Furthermore, we decompose the heterogeneous network into three independent networks that represent the diffusion networks for S,I and *R* to pass through, respectively. Then, we record their corresponding Laplacian matrices as A,B and *C*. By substituting $l_{ij}$ in the original 3N-dimensional ordinary differential equation system (3) with $a_{ij},b_{ij}$ and $c_{ij}$, respectively, we can obtain the SIR rumor propagation system on the heterogeneous network. We must emphasize that system (1) through (4) constitute an original, progressive modeling framework developed in this study. Systems (1), (2) and (3) build upon classical SIR and diffusion theories but incorporate our specific formulations, such as nonlinear incidence and logical growth tailored for rumor dynamics. While considering our most advanced original framework, we utilize specifically designed Laplacian matrices to model a heterogeneous three-layer network and establish the system as follows.

(4) $$\left\{\begin{array}{cc} \frac{dS_{i}}{dt}=d_{1}\sum_{j=1}^{N}a_{ij}S_{j}+rS_{i}\left(1-\frac{S_{i}}{K}\right)-\beta S_{i}I_{i}^{2}-\mu_{1}S_{i},\; & i\in \hat{\Omega },\\ \frac{dI_{i}}{dt}=d_{2}\sum_{j=1}^{N}b_{ij}I_{j}+\beta S_{i}I_{i}^{2}-\mu_{2}I_{i}-\alpha I_{i},\;\;\; & i\in \hat{\Omega },\\ \frac{dR_{i}}{dt}=d_{3}\sum_{j=1}^{N}c_{ij}R_{j}+\alpha I_{i}-\mu_{3}R_{i},\;\;\;\; & i\in \hat{\Omega },\\ S_{i}\left(0\right)>0,I_{i}\left(0\right)⩾,≢0,R_{i}\left(0\right)⩾0,\;\; & i\in \hat{\Omega }. \end{array}\right.$$

The Laplacian matrix in algebra is defined as the difference between the degree and adjacency matrices, representing a semi-positive definite matrix. However, in this paper, it is consistently considered as a semi-negative definite matrix based on our setup. Moving forward, we will provide explicit expressions for A,B and *C*.

$$A=\hat{A}-Diag\left(\sigma_{A}\left(1\right),\sigma_{A}\left(2\right),\cdots \sigma_{A}\left(N\right)\right),$$

$$B=\hat{B}-Diag\left(\sigma_{B}\left(1\right),\sigma_{B}\left(2\right),\cdots \sigma_{B}\left(N\right)\right),$$

$$C=\hat{C}-Diag\left(\sigma_{C}\left(1\right),\sigma_{C}\left(2\right),\cdots \sigma_{C}\left(N\right)\right),$$

where $\sigma_{.}\left(i\right)$ represents the degree of the graph associated with the Laplacian matrix $\left|.\right|$, $\hat{A},\hat{B}$ and $\hat{C}$ represent the adjacency matrix of A,B and *C* respectively.

## 3. Basic Properties

In this section, we determine the conditions for the existence of the positive equilibrium points in System (4). Firstly, the equilibrium points corresponding to the system should be satisfied in the following form:

(5) $$\left\{\begin{array}{c} rS_{i}\left(1-\frac{S_{i}}{K}\right)-\beta S_{i}I_{i}^{2}-\mu_{1}S_{i}=0,\\ \beta S_{i}I_{i}^{2}-\mu_{2}I_{i}-\alpha I_{i}=0,\\ \alpha I_{i}-\mu_{3}R_{i}=0. \end{array}\right.$$

By solving Equation (5), we can derive $S_{i}=\frac{\mu_{2}+\alpha }{\beta I_{i}},R_{i}=\frac{\alpha I_{i}}{\mu_{3}}$ and establish the equilibrium equation

$$f\left(I_{i}\right)≜K\beta^{2}I_{i}^{3}+K\beta \left(\mu_{1}-r\right)I_{i}+r\left(\mu_{2}+\alpha \right)=0.$$

Then, its derivative function is

$$f^{\prime }\left(I_{i}\right)=3K\beta^{2}I_{i}^{2}+K\beta \left(\mu_{1}-r\right).$$

We can observe that $K\beta^{2}>0$ and $K\beta \left(\mu_{1}-r\right)>0$. Thus, when $\mu_{1}⩾r$ (i.e., when the coefficients preceding $I_{i}$ in $f\left(I_{i}\right)$ are greater than or equal to 0), we can infer that $f\left(I_{i}\right)$ never diminishes, thereby precluding the existence of positive roots.

**Figure 2:**
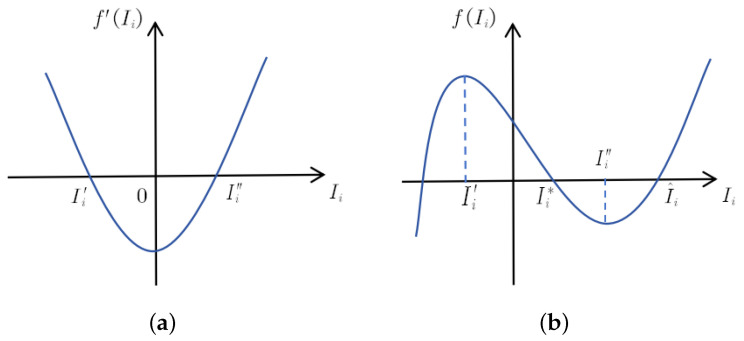
Equilibrium equation $f\left(I_{i}\right)$ and its derivative function.

Next we consider the case of $\mu_{1}<r$. Figure 2a corresponds to the derivative function $f^{\prime }\left(I_{i}\right)$. We denote the roots of $f^{\prime }\left(I_{i}\right)=0$ as $I_{i}^{\prime }$ and $I_{i}^{\prime \prime }$, respectively. Subsequently, it is observed that when $f(I_{i}^{\prime \prime })>0$, the equilibrium equation does not possess positive roots. However, when $f(I_{i}^{\prime \prime })<0$ (as depicted in Figure 2b), positive roots $I_{i}^{*}$ and ${\hat{I}}_{i}$ exist for $f\left(I_{i}\right)=0$.

According to the relationship between $S_{i},I_{i}$ and $R_{i}$, when $I_{i}=I_{i}^{*}$, we can correspondingly determine values for $S_{i}^{*}$ and $R_{i}^{*}$, resulting in the acquirement for the positive equilibrium point $E_{i}^{*}\left(S_{i}^{*},I_{i}^{*},R_{i}^{*}\right)$. Similarly, when $I_{i}={\hat{I}}_{i}$, another positive equilibrium point ${\hat{E}}_{i}\left({\hat{S}}_{i},{\hat{I}}_{i},{\hat{R}}_{i}\right)$ can be obtained. Therefore, we establish the condition for the existence of positive equilibrium points.

**Theorem 1.**  *When $\mu_{1}<r$ and $f(I_{i}^{\prime \prime })<0$, the system exhibits positive equilibrium points $E_{i}^{*}$ and ${\hat{E}}_{i}$.*

Considering that the conditions for the existence of equilibrium points remain consistent, we will select $E_{i}^{*}$ as the representative for further investigation and analysis in this paper.

## 4. Turing Instability

Turing instability refers to a type of symmetry-breaking phenomenon in the spatial dimension, typically characterized by irregular oscillations in the solution across space. The conditions for the emergence of Turing patterns can be summarized as follows: the system exhibits stability against homogeneous disturbances while being unstable to non-homogeneous disturbances.

### 4.1. Stability of the Homogeneous System

In this part, we will employ the conventional research approach of Turing pattern to investigate the stability conditions of the homogeneous system. The Jacobian matrix of the left side in System (5) at the equilibrium point $E_{i}^{*}$ is presented first

$$J\left(E_{i}^{*}\right)=\left(\begin{array}{ccc} r-\frac{2rS_{i}^{*}}{K}-\mu_{1}-\beta {\left(I_{i}^{*}\right)}^{2} & -2\left(\mu_{2}+\alpha \right) & 0\\ \beta {\left(I_{i}^{*}\right)}^{2} & \mu_{2}+\alpha & 0\\ 0 & \alpha & {-\mu }_{3} \end{array}\right).$$

Therefore, through the computation of the subsequent determinant

$$\left|J\left(E_{i}^{*}\right)-\lambda E\right|=\left|\begin{array}{ccc} \lambda -r+\frac{2rS_{i}^{*}}{K}+\mu_{1}+\beta {\left(I_{i}^{*}\right)}^{2} & 2\left(\mu_{2}+\alpha \right) & 0\\ -\beta {\left(I_{i}^{*}\right)}^{2} & \lambda -\mu_{2}-\alpha & 0\\ 0 & -\alpha & \lambda +\mu_{3} \end{array}\right|=0.$$

We can derive the corresponding characteristic equation

(6) $$\lambda^{3}+a_{1}\lambda^{2}+a_{2}\lambda +a_{3}=0,$$

where

(7) $$\begin{array}{cc} a_{1} & =\mu_{3}-\mu_{2}-\alpha +\frac{rS_{i}^{*}}{K},\\ a_{2} & =\left(\mu_{2}+\alpha \right)\left(2r-\mu_{3}-2\mu_{1}-\frac{3rS_{i}^{*}}{K}\right)+\frac{\mu_{3}rS_{i}^{*}}{K},\\ a_{3} & =\mu_{3}\left(\mu_{2}+\alpha \right)\left(2r-2\mu_{1}-\frac{3rS_{i}^{*}}{K}\right). \end{array}$$

According to the Hurwitz criterion, we can obtain the condition that all roots of the third-order characteristic Equation (6) have negative real parts is

$$H_{1}:\left\{\begin{array}{c} \mu_{2}+\alpha -\frac{rS_{i}^{*}}{K}<0,\\ 2r-\frac{3rS_{i}^{*}}{K}-2\mu_{1}>0. \end{array}\right.$$

System (4) can maintain stability in the presence of a small disturbance near the homogeneous equilibrium point, given that condition $H_{1}$ is satisfied.

In the conventional derivation process of Turing instability, we expand the perturbation in the eigenspace of the Laplacian matrix to obtain a lower-order characteristic equation of the system. For the modulus k=0, the eigenvalues of the characteristic equation exhibit negative real parts, while for the modulus k>0, the eigenvalues have positive real parts. However, on homogeneous networks with large network sizes, employing traditional methods may not facilitate determining parameters for Turing pattern occurrence. On heterogeneous networks, where perturbations can not be expanded in terms of the feature space provided by the Laplace matrix, traditional approaches fail and we should find alternative solutions.

### 4.2. The Necessary Condition for Turing Instability on Different Networks

#### 4.2.1. Preliminaries

To facilitate the discussion, let us begin by establishing some assumptions and definitions [33]. The eigenvalues of matrices A,B and *C* are denoted as $\theta_{n},\delta_{n},\eta_{n}\left(n=1,2\cdots N\right)$. For any square matrix *L* of order *N*, its spectral bounds $s\left(L\right)$ and spectral radius $p\left(L\right)$ can be defined as follows:

sL=maxiReλi,pL=maxiλi.

The fact that A,B,C are symmetric matrices, in conjunction with Perron–Frobenius theory, implies that $\theta_{n},\delta_{n},\eta_{n}$ are non-positive real numbers. Firstly, we present the Jacobian matrix *J* for System (4) at the equilibrium point $E_{i}^{*}$

$$J=\left(\begin{array}{ccc} d_{1}A+\left(r-\frac{2rS_{i}^{*}}{K}-\mu_{1}-\beta {\left(I_{i}^{*}\right)}^{2}\right)E_{N} & -2\left(\mu_{2}+\alpha \right)E_{N} & 0_{N}\\ \beta {\left(I_{i}^{*}\right)}^{2}E_{N} & d_{2}B+\left(\mu_{2}+\alpha \right)E_{N} & 0_{N}\\ 0_{N} & \alpha E_{N} & d_{3}C-\mu_{3}E_{N} \end{array}\right).$$

The calculation of $\left|J-\lambda E_{3N}\right|=0$ enables us to obtain

$$\left|\begin{array}{ccc} d_{1}A+\left(r-\frac{2rS_{i}^{*}}{K}-\mu_{1}-\beta {\left(I_{i}^{*}\right)}^{2}-\lambda \right)E_{N} & -2\left(\mu_{2}+\alpha \right)E_{N} & 0_{N}\\ \beta {\left(I_{i}^{*}\right)}^{2}E_{N} & d_{2}B+\left(\mu_{2}+\alpha -\lambda \right)E_{N} & 0_{N}\\ 0_{N} & \alpha E_{N} & d_{3}C-\left(\mu_{3}+\lambda \right)E_{N} \end{array}\right|=0.$$

Since *C* is a real symmetric matrix, there exists an orthogonal matrix *Q* that

$$QCQ^{T}=\left(\begin{array}{cccc} \eta_{1} & & & \\ & \eta_{2} & & \\ & & \ddots & \\ & & & \eta_{N} \end{array}\right)≜C^{*}.$$

According to $\left|Q\right|\neq 0$, we can obtain

$$\left|\left(\begin{array}{ccc} Q & 0_{N} & 0_{N}\\ 0_{N} & Q & 0_{N}\\ 0_{N} & 0_{N} & Q \end{array}\right)\left(J-\lambda E_{3N}\right)\left(\begin{array}{ccc} Q^{T} & 0_{N} & 0_{N}\\ 0_{N} & Q^{T} & 0_{N}\\ 0_{N} & 0_{N} & Q^{T} \end{array}\right)\right|=0.$$

The result is obtained through the calculation for expansion

(8) $$\begin{array}{cc} \; & \left|\begin{array}{ccc} d_{1}QAQ^{T}+\left(r-\frac{2rS_{i}^{*}}{K}-\mu_{1}-\beta {\left(I_{i}^{*}\right)}^{2}-\lambda \right)E_{N} & -2\left(\mu_{2}+\alpha \right)E_{N} & 0_{N}\\ \beta {\left(I_{i}^{*}\right)}^{2}E_{N} & d_{2}QBQ^{T}+\left(\mu_{2}+\alpha -\lambda \right)E_{N} & 0_{N}\\ 0_{N} & \alpha E_{N} & d_{3}C^{*}-\left(\mu_{3}+\lambda \right)E_{N} \end{array}\right|\\ = & \left|d_{3}C^{*}-\left(\mu_{3}+\lambda \right)E_{N}\right|\left|\begin{array}{cc} d_{1}QAQ^{T}+\left(r-\frac{2rS_{i}^{*}}{K}-\mu_{1}-\beta {\left(I_{i}^{*}\right)}^{2}-\lambda \right)E_{N} & -2\left(\mu_{2}+\alpha \right)E_{N}\\ \beta {\left(I_{i}^{*}\right)}^{2}E_{N} & d_{2}QBQ^{T}+\left(\mu_{2}+\alpha -\lambda \right)E_{N} \end{array}\right|\\ = & 0. \end{array}$$

The assumption is made that $d_{3}C^{*}-\left(\mu_{3}+\lambda \right)E_{N}$ is irreversible; then, we have

$$\left|\begin{array}{cccc} d_{3}\eta_{1}-\left(\mu_{3}+\lambda \right) & 0 & \cdots & 0\\ 0 & d_{3}\eta_{2}-\left(\mu_{3}+\lambda \right) & \cdots & 0\\ \vdots & \vdots & \ddots & \vdots \\ 0 & 0 & \cdots & d_{3}\eta_{N}-\left(\mu_{3}+\lambda \right) \end{array}\right|=0.$$

In other words, there exists $\eta_{n}\in \left(\eta_{1},\eta_{2},\cdots \eta_{N}\right)$ that $d_{3}\eta_{n}-\left(\mu_{3}+\lambda \right)=0$; thus, $\lambda =d_{3}\eta_{n}-\mu_{3}<0$ leads to a contradiction with the presence of a positive real part of λ. Consequently, considering in conjunction with (8) necessitates the following equation to be true:

(9) $$\left|\begin{array}{cc} d_{1}QAQ^{T}+\left(r-\frac{2{rS_{i}}^{*}}{K}-\mu_{1}-\beta {\left({I_{i}}^{*}\right)}^{2}-\lambda \right)E_{N} & -2\left(\mu_{2}+\alpha \right)E_{N}\\ \beta {\left({I_{i}}^{*}\right)}^{2}E_{N} & d_{2}QBQ^{T}+\left(\mu_{2}+\alpha -\lambda \right)E_{N} \end{array}\right|=0.$$

#### 4.2.2. Homogeneous Networks

When considering homogeneous networks, where Laplacian matrices A,B and *C* are all identical, we have A=B=C. That is to say, $QAQ^{T}=QBQ^{T}=C^{*}$. Thus, Equation (9) can be reformulated in the following form:

$$\left|\begin{array}{cc} \underset{n}{\mathrm{diag}}\left\{d_{1}\eta_{n}+r-\frac{2rS_{i}^{*}}{K}-\mu_{1}-\beta {\left(I_{i}^{*}\right)}^{2}-\lambda \right\} & -2\left(\mu_{2}+\alpha \right)E_{N}\\ \beta {\left(I_{i}^{*}\right)}^{2}E_{N} & \underset{n}{\mathrm{diag}}\left\{d_{2}\eta_{n}+\mu_{2}+\alpha -\lambda \right\} \end{array}\right|=0.$$

After elementary row change, we can obtain

$$\prod_{n=1}^{N}\left|\begin{array}{cc} \underset{n}{\mathrm{diag}}\left\{d_{1}\eta_{n}+r-\frac{2{rS_{i}}^{*}}{K}-\mu_{1}-\beta {\left({I_{i}}^{*}\right)}^{2}-\lambda \right\} & -2\left(\mu_{2}+\alpha \right)E_{N}\\ \beta {\left({I_{i}}^{*}\right)}^{2}E_{N} & \underset{n}{\mathrm{diag}}\left\{d_{2}\eta_{n}+\mu_{2}+\alpha -\lambda \right\} \end{array}\right|=0,$$

and then we have the corresponding characteristic equation $\lambda^{2}+u\lambda +v=0,$ where

(10) $$\begin{array}{cc} u= & -d_{1}\eta_{n}-r+\frac{2{rS_{i}}^{*}}{K}+\mu_{1}+\beta {\left({I_{i}}^{*}\right)}^{2}-d_{2}\eta_{n}-\mu_{2}-\alpha \\ = & -\left(d_{1}+d_{2}\right)\eta_{n}+\frac{{rS_{i}}^{*}}{K}-\mu_{2}-\alpha ,\\ v= & d_{1}d_{2}\eta_{n}^{2}+\left(d_{1}\mu_{2}+d_{1}\alpha +d_{2}r-\frac{2rd_{2}{S_{i}}^{*}}{K}-d_{2}\mu_{1}-d_{2}\beta {\left({I_{i}}^{*}\right)}^{2}\right)\eta_{n}\\ \; & +\mu_{2}r+\alpha r-\frac{2r\mu_{2}{S_{i}}^{*}}{K}-\frac{2{r\alpha S_{i}}^{*}}{K}-\mu_{1}\mu_{2}-\mu_{1}\alpha +\mu_{2}\beta {\left({I_{i}}^{*}\right)}^{2}+\alpha \beta {\left({I_{i}}^{*}\right)}^{2}\\ = & d_{1}d_{2}\eta_{n}^{2}+\left[d_{1}\left(\mu_{2}+\alpha \right)-\frac{rd_{2}{S_{i}}^{*}}{K}\right]\eta_{n}+\left(\mu_{2}+\alpha \right)\left(2r-\frac{3{rS_{i}}^{*}}{K}-2\mu_{1}\right). \end{array}$$

The assumption is that $\lambda_{1}$ and $\lambda_{2}$ are two eigenvalues. If there exists a positive real part, then in accordance with Veda’s theorem, we obtain $\lambda_{1}+\lambda_{2}>0$ or $\lambda_{1}\lambda_{2}<0$. Commencing with the initial case $\lambda_{1}+\lambda_{2}>0$, we can deduce $\left(d_{1}+d_{2}\right)\eta_{n}-\frac{rS_{i}^{*}}{K}+\mu_{2}+\alpha >0.$ When considering the case $\lambda_{1}\lambda_{2}<0$, we have

$$g\left(\eta_{n}\right)≜d_{1}d_{2}\eta_{n}^{2}+\left[d_{1}\left(\mu_{2}+\alpha \right)-d_{2}\frac{rS_{i}^{*}}{K}\right]\eta_{n}+\left(\mu_{2}+\alpha \right)\left(2r-\frac{3rS_{i}^{*}}{K}-2\mu_{1}\right)<0.$$

We assume that $g\left(\eta_{n}\right)$ is a quadratic equation in terms of $\eta_{n}$, and $\eta_{1}^{*}$ and $\eta_{2}^{*}$ are two roots of the equation $g\left(\eta_{n}\right)=0$. Subsequently, when the following inequality holds

$$\left\{\begin{array}{c} d_{1}\left(\mu_{2}+\alpha \right)-\frac{rd_{2}S_{i}^{*}}{K}>0,\\ {\left[d_{1}\left(\mu_{2}+\alpha \right)-\frac{rd_{2}S_{i}^{*}}{K}\right]}^{2}-4d_{1}d_{2}\left(\mu_{2}+\alpha \right)\left(2r-\frac{3rS_{i}^{*}}{K}-2\mu_{1}\right)>0, \end{array}\right.$$

the existence of $\eta_{n}\in \left(\eta_{1}^{*},\eta_{2}^{*}\right)$ can be obtained to prove $\lambda_{1}\lambda_{2}<0$. Considering the condition $H_{1}$ in combination, a contradiction $\left(d_{1}+d_{2}\right)\eta_{n}-\frac{rS_{i}^{*}}{K}+\mu_{2}+\alpha >0$ is identified within the case $\lambda_{1}+\lambda_{2}>0$. Therefore, it will be omitted. In conclusion, we can establish the necessary condition for Turing instability to manifest in the homogeneous networks as

$$\left\{\begin{array}{c} \mu_{2}+\alpha -\frac{rS_{i}^{*}}{K}<0,\\ d_{1}\left(\mu_{2}+\alpha \right)-d_{2}\frac{rS_{i}^{*}}{K}>0,\\ {\left[d_{1}\left(\mu_{2}+\alpha \right)-\frac{rd_{2}S_{i}^{*}}{K}\right]}^{2}-4d_{1}d_{2}\left(\mu_{2}+\alpha \right)\left(2r-\frac{3rS_{i}^{*}}{K}-2\mu_{1}\right)>0,\\ 2r-\frac{3rS_{i}^{*}}{K}-2\mu_{1}>0. \end{array}\right.$$

#### 4.2.3. Heterogeneous Networks

In heterogeneous networks, Laplacian matrices A,B and *C* are not equal. It is evident that the three Laplacian matrices can not undergo unitary transformation simultaneously. Therefore, based on ref. [33], we will employ matrix spectrum theory to derive the necessary conditions for Turing instability.

Firstly, Equation (9) can be transformed into the following form by assuming Reλ=x and Imλ=y:

(11) $$\left|\begin{array}{cc} d_{1}QAQ^{T}+\left(r-\frac{2rS_{i}^{*}}{K}-\mu_{1}-\beta {\left(I_{i}^{*}\right)}^{2}-x-iy\right)E_{N} & -2\left(\mu_{2}+\alpha \right)E_{N}\\ \beta {\left(I_{i}^{*}\right)}^{2}E_{N} & d_{2}QBQ^{T}+\left(\mu_{2}+\alpha -x-iy\right)E_{N} \end{array}\right|=0.$$

Since $QAQ^{T}$ is a real symmetric matrix, there exists an orthogonal matrix $Q^{\prime }$ such that $\left(Q^{\prime }Q\right)A{\left(Q^{\prime }Q\right)}^{T}$ becomes a diagonal matrix. Consequently, $d_{1}QAQ^{T}+\left(r-\frac{2rS_{i}^{*}}{K}-\mu_{1}-\beta {\left(I_{i}^{*}\right)}^{2}-x-iy\right)E_{N}$ can be transformed into a diagonal matrix $\underset{m}{\mathrm{diag}}\left\{d_{1}\theta_{m}+r-\frac{2rS_{i}^{*}}{K}-\mu_{1}-\beta {\left(I_{i}^{*}\right)}^{2}-x-iy\right\}$, where the eigenvalue is $x_{n}+iy_{n},n\in \left\{1,2,\cdots N\right\}$. Given the fact that $d_{1}QAQ^{T}+\left(r-\frac{2rS_{i}^{*}}{K}-\mu_{1}-\beta {\left(I_{i}^{*}\right)}^{2}-x-iy\right)E_{N}$, $d_{2}QBQ^{T}$ and $\left(\mu_{2}+\alpha -x-iy\right)E_{N}$ are all normal matrices, according to the corollary in ref. [33], the necessary conditions can be obtained as follows.

(12) $$\begin{array}{cc} \; & \left[{\left(d_{1}\theta_{m}+r-\frac{2rS_{i}^{*}}{K}-\mu_{1}-\beta {\left(I_{i}^{*}\right)}^{2}-x\right)}^{2}+y^{2}\right]\left(\mu_{2}+\alpha -x\right)\\ + & 2\beta {\left(I_{i}^{*}\right)}^{2}\left(\mu_{2}+\alpha \right)\left(d_{1}\theta_{m}+r-\frac{2rS_{i}^{*}}{K}-\mu_{1}-\beta {\left(I_{i}^{*}\right)}^{2}-x\right)⩾0. \end{array}$$

There exists $m\in \left\{1,2,\cdots N\right\}$ such that Inequation (12) possesses solutions for x>0. In addition, we get Inequality (13) by scaling (12) and obtain the following results:

(13) $$\begin{array}{cc} \; & \left[{\left(d_{1}\theta_{m}+r-\frac{2rS_{i}^{*}}{K}-\mu_{1}-\beta {\left(I_{i}^{*}\right)}^{2}-x\right)}^{2}+y^{2}\right]\left(\mu_{2}+\alpha \right)\\ + & 2\beta {\left(I_{i}^{*}\right)}^{2}\left(\mu_{2}+\alpha \right)\left(d_{1}\theta_{m}+r-\frac{2rS_{i}^{*}}{K}-\mu_{1}-\beta {\left(I_{i}^{*}\right)}^{2}-x\right)⩾0. \end{array}$$

Next, according to any norm of a consistent matrix that is greater than the spectral radius, we give the following inequality

(14) $$\begin{array}{cc} \left|\lambda \right|= & \sqrt{x^{2}+y^{2}}⩽P\left(J\right)⩽{∥J∥}_{2}\\ ⩽ & {∥\left(\begin{array}{ccc} d_{1}A+\left(r-\frac{2rS_{i}^{*}}{K}-\mu_{1}-\beta {\left(I_{i}^{*}\right)}^{2}\right)E_{N} & 0_{N} & 0_{N}\\ 0_{N} & d_{2}B+\left(\mu_{2}+\alpha \right)E_{N} & 0_{N}\\ 0_{N} & 0_{N} & d_{3}C-\mu_{3}E_{N} \end{array}\right)∥}_{2}\\ \; & +{∥\left(\begin{array}{ccc} 0_{N} & -2\left(\mu_{2}+\alpha \right)E_{N} & 0_{N}\\ \beta {\left(I_{i}^{*}\right)}^{2}E_{N} & 0_{N} & 0_{N}\\ 0_{N} & \alpha E_{N} & 0_{N} \end{array}\right)∥}_{2}\\ = & \max_{n}\left\{\left|d_{1}\theta_{n}+r-\frac{2rS_{i}^{*}}{K}-\mu_{1}-\beta {\left(I_{i}^{*}\right)}^{2}\right|,\left|d_{2}\delta_{n}+\mu_{2}+\alpha \right|,\mu_{3}-d_{3}\eta_{n}\right\}\\ \; & +\max_{n}\left\{\beta {\left(I_{i}^{*}\right)}^{2},\sqrt{4{\left(\mu_{2}+\alpha \right)}^{2}+\alpha^{2}}\right\}≜\varphi . \end{array}$$

Thus, Inequality (13) can be rewritten as

(15) $$\begin{array}{cc} \; & \left[{\left(d_{1}\theta_{m}+r-\frac{2rS_{i}^{*}}{K}-\mu_{1}-\beta {\left(I_{i}^{*}\right)}^{2}\right)}^{2}-2x\left(d_{1}\theta_{m}+r-\frac{2rS_{i}^{*}}{K}-\mu_{1}-\beta {\left(I_{i}^{*}\right)}^{2}\right)+\varphi^{2}\right]\left(\mu_{2}+\alpha \right)\\ + & 2\beta {\left(I_{i}^{*}\right)}^{2}\left(\mu_{2}+\alpha \right)\left(d_{1}\theta_{m}+r-\frac{2rS_{i}^{*}}{K}-\mu_{1}-\beta {\left(I_{i}^{*}\right)}^{2}-x\right)⩾0. \end{array}$$

For the simplicity of discussion, let us assume that $q=d_{1}\theta_{m}+r-\frac{2rS_{i}^{*}}{K}-\mu_{1}-\beta {\left(I_{i}^{*}\right)}^{2}$, thereby enabling us to derive

$$\left(q^{2}+\varphi^{2}\right)\left(\mu_{2}+\alpha \right)-2xq\left(\mu_{2}+\alpha \right)+2\beta q{\left(I_{i}^{*}\right)}^{2}\left(\mu_{2}+\alpha \right)-2x\beta {\left(I_{i}^{*}\right)}^{2}\left(\mu_{2}+\alpha \right)⩾0.$$

The left half of the above inequality is expressed as a first-order function with respect to *x*. Let

$$\phi_{m}\left(x\right)=b_{1}\left(\theta_{m}\right)x+b_{2}\left(\theta_{m}\right),$$

where

(16) $$\begin{array}{cc} b_{1}\left(\theta_{m}\right)= & -2q\left(\mu_{2}+\alpha \right)-2\beta {\left(I_{i}^{*}\right)}^{2}\left(\mu_{2}+\alpha \right)\\ = & -2\left(\mu_{2}+\alpha \right)\left(d_{1}\theta_{m}+r-\frac{2rS_{i}^{*}}{K}-\mu_{1}\right),\\ b_{2}\left(\theta_{m}\right)= & \left(q^{2}+\varphi^{2}\right)\left(\mu_{2}+\alpha \right)+2\beta q{\left(I_{i}^{*}\right)}^{2}\left(\mu_{2}+\alpha \right)\\ = & \left(\mu_{2}+\alpha \right)\left[\varphi^{2}-\left(d_{1}\theta_{m}-\frac{rS_{i}^{*}}{K}\right)\left(d_{1}\theta_{m}+2r-\frac{3rS_{i}^{*}}{K}-2\mu_{1}\right)\right]. \end{array}$$

If $b_{1}\left(\theta_{m}\right)>0$ holds, there must exist $m\in \left\{1,2,\cdots N\right\}$ such that $\phi_{m}\left(x\right)>0$. That is to say, we need the following condition to be satisfied:

$$d_{1}\theta_{m}+r-\frac{2rS_{i}^{*}}{K}-\mu_{1}>0.$$

If $b_{1}\left(\theta_{m}\right)<0$ holds, then we can derive $b_{2}\left(\theta_{m}\right)>0$. In other words, the following requirements must be fulfilled

$$\left\{\begin{array}{c} d_{1}\theta_{m}+r-\frac{2rS_{i}^{*}}{K}-\mu_{1}<0,\\ \varphi^{2}-\left(d_{1}\theta_{m}-\frac{rS_{i}^{*}}{K}\right)\left(d_{1}\theta_{m}+2r-\frac{3rS_{i}^{*}}{K}-2\mu_{1}\right)>0. \end{array}\right.$$

The necessary conditions for the occurrence of Turing instability on the heterogeneous networks can be identified at this point

$$H_{2}:\left\{\begin{array}{c} \mu_{2}+\alpha -\frac{rS_{i}^{*}}{K}<0,\\ 2r-\frac{3rS_{i}^{*}}{K}-2\mu_{1}>0,\\ d_{1}\theta_{m}+r-\frac{2rS_{i}^{*}}{K}-\mu_{1}>0. \end{array}\right.$$

or

$$H_{3}:\left\{\begin{array}{c} \mu_{2}+\alpha -\frac{rS_{i}^{*}}{K}<0,\\ 2r-\frac{3rS_{i}^{*}}{K}-2\mu_{1}>0,\\ d_{1}\theta_{m}+r-\frac{2rS_{i}^{*}}{K}-\mu_{1}>0,\\ \varphi^{2}-\left(d_{1}\theta_{m}-\frac{rS_{i}^{*}}{K}\right)\left(d_{1}\theta_{m}+2r-\frac{3rS_{i}^{*}}{K}-2\mu_{1}\right)>0. \end{array}\right.$$

The following provides supplementary explanations for the meanings of (H2) and (H3) in terms of their practical significance. For (H2), considering the specific meanings of the parameters, the first equation requires that the replacement rate of spreaders be less than the net growth advantage of the susceptible population. The second equation requires that the environmental carrying capacity should be as large as possible. Equation (3) requires that the susceptible population should not be lost too quickly. Only when the susceptible population is active enough can a clustering pattern be formed. For (H3), the first three inequalities correspond to the dynamical conditions of (H2), and the complex expression in the fourth equation characterizes the intricate interaction between the network structure and the diffusion behavior. Under the premise that the parameters satisfy the inequalities, when substituted into the system, the rumor propagation state can be captured through Turing patterns.

## 5. Numerical Simulations

The correctness of certain conclusions in Section 4 will be verified through numerical simulation in this section. All numerical simulations are implemented using MATLAB R2022a, employing a forward Euler finite-difference scheme adapted for discrete Laplacian matrices to carry out time integration. Furthermore, to provide an intuitive understanding of the abstract patterns, we introduce a university campus forum where a specific rumor begins to spread. Here, nodes represent individual users and edges represent their social connections. In addition, we will explore more effective control measures to suppress the propagation of rumors by analyzing patterns across different networks.

### 5.1. The Case of Homogeneous Networks

First, we examine the characteristics of the Turing patterns on the homogeneous networks. In the homogeneous network, we regard the transmission networks of the three groups of people $\left(S,I,R\right)$ as a three-layer network structure. The first layer, the diffusion network of the susceptible individuals, represents the channels through which unexposed susceptible individuals come into contact with the rumor information, such as public information dissemination platforms; the second layer, the diffusion network of the spreaders, represents the channels through which active rumor spreaders disseminate information; the third layer, the diffusion network of the immune individuals, represents the channels through which genuine informed users or official accounts disseminate information, such as government official accounts or scientific communication communities. Based on this network architecture, we explored the diffusion states under changes in specific parameters and the number of contact individuals.

**Figure 3:**
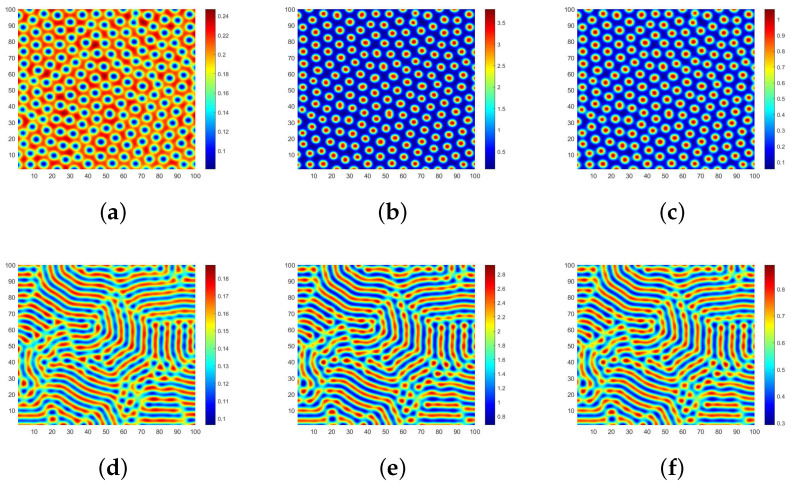
Different shapes of Turing patterns with r=0.26 and r=0.4.

The parameter selection is strictly constrained by practical meanings and the mathematical requirements derived in the previous sections. According to Table 1, parameters representing transition and replacement rates typically fall within the range of (0,1]. More importantly, the chosen values must strictly ensure the existence of a positive equilibrium point (e.g., $\mu_{1}<r$) and satisfy the necessary conditions for Turing instability. Therefore, we opt for $K=0.5,\beta =0.8,\mu_{1}=0.4,\mu_{2}=0.1,\mu_{3}=0.3,\alpha =0.1,d_{1}=10,d_{2}=0.1,$ $d_{3}=0.1$. Figure 3 investigates the case where the three-layer network structure is quadrilateral lattice (QL) networks, illustrating how variations in the natural growth rate *r* of different individuals impact the pattern morphology.

When r=2.6, the spot patterns depicted in Figure 3a–c represent the distribution of S,I and *R*, respectively. The blue region in Figure 3a represents the high-density area where ignorant individuals gather, while the red region in Figure 3b,c signifies the high-density area where spreaders and immune individuals congregate. When the natural growth rate *r* is low, spreaders tend to restrict their interactions to those in close proximity via chat software platforms, forming isolated clusters. This highly localized clustering aligns with real-world patterns of network rumor propagation, suggesting that large-scale outbreaks are unlikely during the initial stages. Consequently, such insight facilitates the implementation of IP blocking policies targeting specific regional clusters to contain the rumor. Furthermore, we observe that each blue spot is surrounded by green areas along its edges, indicating that large-scale outbreaks of Internet rumors are unlikely during early propagation stages. Hence, if authorities promptly adopt a policy of refuting rumors, it can prevent ignorant individuals from transitioning into spreaders and effectively curtail rumor spread.

When r=4, the patterns which are formed in the coexistence of spots and stripes from Figure 3d–f exhibit the distribution of S,I,R in space. Based on a longitudinal comparison with Figure 3a–c, the conventional spatial distribution of ignorant individuals is disrupted and forms clusters in spots and stripes. Furthermore, the spreader and immune individuals are no longer confined to fixed areas but flow freely in space. Intuitively, within the context of our university campus forum toy example, this indicates that the rumor has broken out of localized social connections and is spreading more widely across the network. We have observed significant changes in the spatial distribution patterns of all types of individuals. As the natural growth rate *r* of ignorant individuals increases, the spreaders are no longer satisfied with the initial purpose of propagation and spread the rumors in groups of the same city. Consequently, the corresponding propagation area expands both in size and diameter range, posing greater challenges for rumor control.

**Figure 4:**
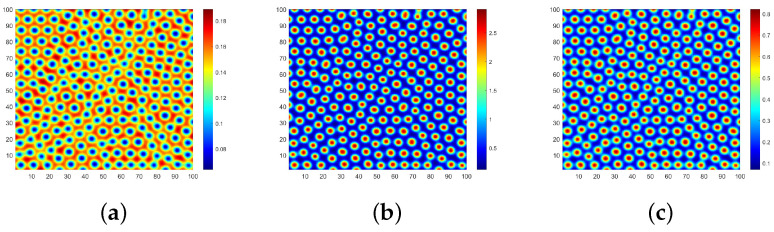
Different shapes of Turing patterns with β=1.3.

The natural growth rate, *r* can describe the initial rate of rumor spread, but it lacks description of the overall change in the speed of spread. Therefore, we use the infection rate β as the key indicator. At this time, we modify β to 1.3. By changing the infection rate to simulate the process of changing the speed of spread, we draw the corresponding Turing patterns under the same parameters and compare them with Figure 3a–c. Observing Figure 4a–c, we find that the basic distribution patterns of the population remain consistent; that is, they are all point-like patterns. The difference is that the maximum value of the corresponding population has decreased. This may be because, during the rumor spread process, as the transmission rate increases, the nonlinear term strengthens, and the spread process becomes more intense, so the susceptible individuals are quickly “consumed”, and the overall scale undergoes a certain reduction. Under the premise of a reduced susceptible population size, the number of infected individuals naturally decreases, and the population gathers at a slightly lower density.

After performing calculations, we have determined that the aforementioned parameters satisfy the necessary conditions for Turing instability in homogeneous networks. In Figure 3, we show the specific existence of Turing patterns when the three-layer network structure is a QL network through the spatial domain. Subsequently, we will employ spectral domain analysis to validate our findings.

**Figure 5:**
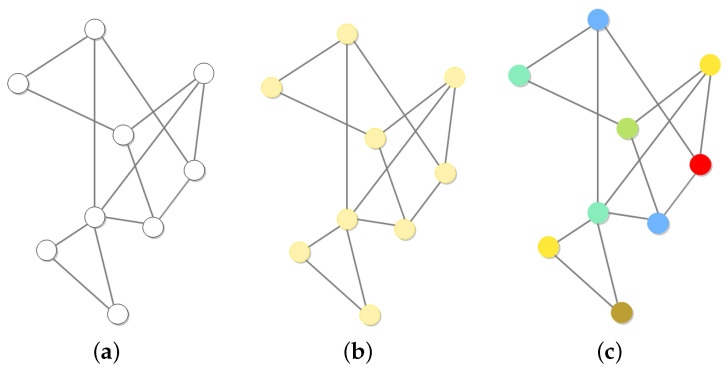
Schematic of different network structures.

The fully connected network structure of the susceptible individual *S* in Figure 5a consists of *N* nodes. If we consider the network structure as a vector, it can be represented in terms of ${\left(\begin{array}{cccc} S_{1} & S_{2} & \cdots & S_{N} \end{array}\right)}^{T}$, which belongs to the $R^{N}$ space. Assuming that the eigenvalues of the Laplace matrix *A* are $\theta_{1},\theta_{2},\cdots \theta_{N}$, and their corresponding eigenvectors are $\gamma_{1},\gamma_{2},\cdots \gamma_{N}$ respectively, we can express the vector linearly as

$$\left(\begin{array}{c} S_{1}\\ S_{2}\\ \vdots \\ S_{N} \end{array}\right)=k_{1}\gamma_{1}+k_{2}\gamma_{2}+\cdots +k_{N}\gamma_{N},$$

where $k_{1},k_{2},\cdots k_{N}$ represent the Fourier coefficients. Due to the non-full rank of the Laplace matrix *A*, there must exist a scenario where the eigenvalue is 0 and its corresponding eigenvector of order *n* contains ${\left(\begin{array}{cccc} 1 & 1 & \cdots & 1 \end{array}\right)}^{T}$. For the sake of narration, we assume the vector ${\left(\begin{array}{cccc} 1 & 1 & \cdots & 1 \end{array}\right)}^{T}$ as $\gamma_{1}$. Based on the fully connected network structure setting, it can be deduced that there is at most one eigenvalue of 0. By utilizing Perron–Frobenius theory, we can conclude that the eigenvector associated with 0 eigenvalue must be $\gamma_{1}$. Considering the homogeneous case illustrated in Figure 5b, where all nodes possess identical values, then

$$\left(\begin{array}{c} S_{1}\\ S_{2}\\ \vdots \\ S_{N} \end{array}\right)=\left(\begin{array}{c} S_{i}^{\prime }\\ S_{i}^{\prime }\\ \vdots \\ S_{i}^{\prime } \end{array}\right)=k_{1}\gamma_{1}+k_{2}\gamma_{2}+\cdots +k_{N}\gamma_{N}=k_{1}\left(\begin{array}{c} 1\\ 1\\ \vdots \\ 1 \end{array}\right),$$

$S_{i}^{\prime }$ stands for any value in $S_{1},S_{2},\cdots S_{N}$. The network structure can be represented as a non-zero integer multiple (Fourier coefficient $k_{1}$) of the positive eigenvector corresponding to 0 eigenvalue in the Laplacian matrix *A*. In other words, for the homogeneous case, only Fourier coefficients corresponding to the 0 eigenvalue can have non-zero values. Depicted in Figure 5c is in the non-homogeneous case, where nodes possess distinct values

$$\left(\begin{array}{c} S_{1}\\ S_{2}\\ \vdots \\ S_{N} \end{array}\right)=k_{1}\gamma_{1}+k_{i}\gamma_{i},$$

where $i\in \left(2,3,\cdots N\right)$. That is to say, for the non-homogeneous case, apart from the 0 eigenvalue corresponding to the Fourier coefficient being non-zero, there must also be other non-zero Fourier coefficients.

**Figure 6:**
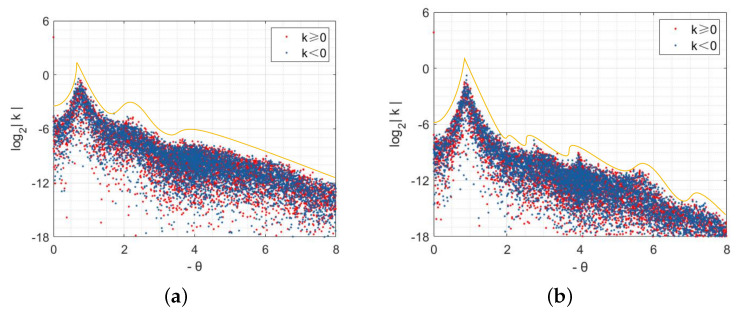
Distribution of coefficients after Fourier expansion of *S* in regular networks.

According to the theoretical analysis presented above, as shown in Figure 6a,b, we depict the spectral domain of Figure 3a,d. In order to ensure that the scattered points are distributed exclusively in the right half plane of the coordinate axis, we assign negative eigenvalues to the abscissa, and the ordinate represents the logarithm of the absolute value of the Fourier coefficient with base 2. In order to emphasize significant features of the figure, only Fourier coefficients with absolute values greater than $2^{-18}$ are displayed in Figure 6. Upon observation, it can be noted that scattered points are primarily concentrated near the yellow curve, exhibiting an overall trend of initial increase followed by decrease.

**Figure 7:**
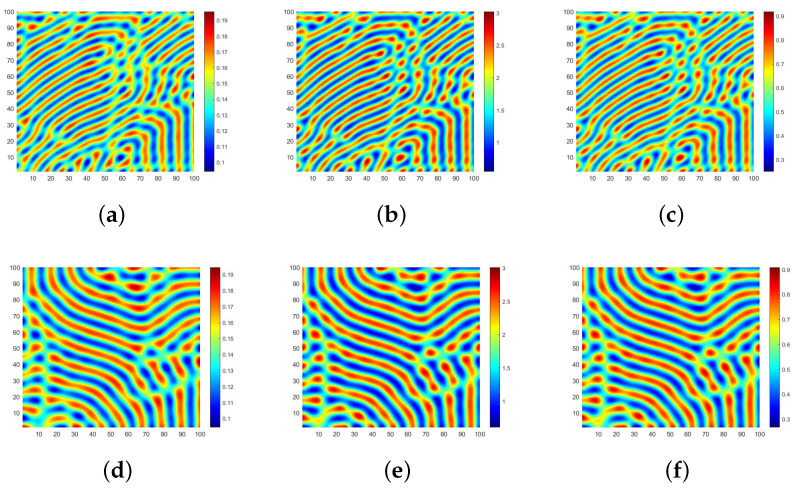
The influence of different regular networks for the patterns.

Specifically, in Figure 7a, when *r* equals 0.26, it becomes evident that the ordinate formed by scatter points exceeds −6. By examining the yellow curve, we observe that scatter points distribute within an abscissa range from 0 to 2, forming a “sharp angle”. Additionally, certain positions along this curve exhibit a “concave–convex” shape indicating fluctuations in corresponding Fourier coefficients. As *r* increases to 0.4 (as depicted in Figure 7b), there is a drop in ordinate for scatter points’ initial position, which leads to the distribution of scatter points becoming sparse within the “sharp angle”. Furthermore, through changes observed in the yellow curve, it becomes apparent that the overall fluctuation in Fourier coefficient becomes more pronounced.

The analysis in Figure 3 focuses on the case when each node in the regular network has a degree of 4. Subsequently, we select the values of parameters corresponding to Figure 3d and increase the degree of each node in the network to 6 and 8, respectively. Figure 7 is utilized to further investigate the influence on the spatial distribution of individuals with the alterations in network structures.

The spatial distribution of S,I,R in the hexagonal lattice network (QL) is illustrated in Figure 7a–c. Upon observation, it can be noted that the overall pattern primarily consists of spots. However, when comparing Figure 7a to Figure 3d, the pattern appears elongated in the upper-right and lower-left directions. Compared with Figure 3e,f, the situations in Figure 7b,c are also identical. Figure 7d–f explore a scenario where all three layers of networks are octagonal lattice (OL) networks. Compared with the distribution of different individuals in QL networks, it becomes evident that the shapes of spots have significantly diminished, and individuals of all types tend to cluster together, forming stripe-like structures in their spatial distribution. Clearly, we can conclude that different network structures within the regular network do not alter Turing pattern shapes but rather influence the spatial distribution patterns of populations.

In fact, in a regular network with increased connecting edges between network nodes, i.e., more channels for individuals to circulate, it facilitates spreading in rumor propagation and leads to a wider spread range. This phenomenon affects the spatial distribution of populations to some extent and escalates the difficulty of rumor control. In today’s society, Xiaohongshu, Weibo and other social platforms are emerging as mainstream platforms. Considering each social platform as a node within the network, when individuals’ cognition for quantity of social platforms expands, it is obvious that the circulation of populations between platforms will increase, which intensifies the risk of rumor propagation. Therefore, implementing restrictions on new platform users such as adopting stricter manual review methods for their published information would contribute to effective rumor control.

In a regular network, it is certain whether there exists a connected edge between any two nodes, but in reality, the movement of individuals from one node to another is not an inevitable phenomenon where it occurs with a specific probability. Therefore, solely considering the regular network fails to explain the randomness observed in diffusion processes. To restore the characteristics of real networks, we will now investigate how these changes contribute to the irregular network structures.

**Figure 8:**
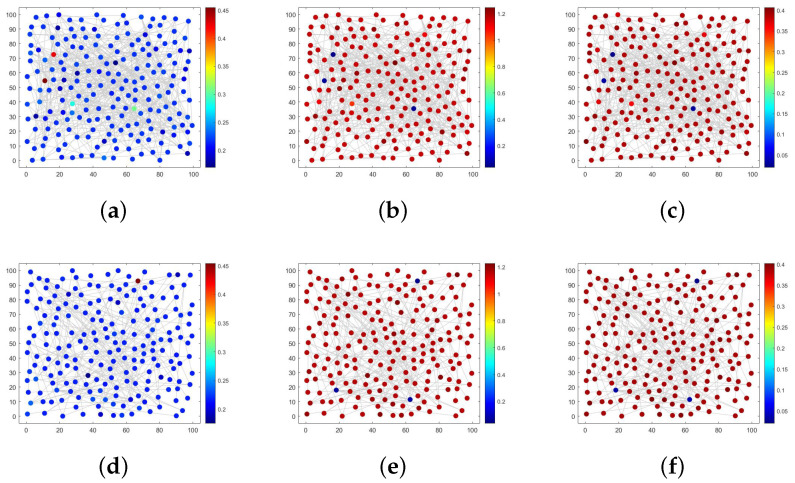
Turing patterns in ER and BA networks.

An ER random network effectively illustrates that the decision to join a social network platform is driven by individual preferences across various populations. Assuming r=0.26, $K=0.5,\beta =0.8,\mu_{1}=0.4,\mu_{2}=0.1,\mu_{3}=0.3,\alpha =0.1,d_{1}=10.d_{2}=0.1,d_{3}=0.1$, and the probability of connecting edges is 0.02. As shown in Figure 8a, the majority of nodes exhibit a dark blue color, and few of the ignorant individuals are concentrated in this part of nodes. The remaining nodes show light green or dark red; thus, the ignorant individuals are dominant in these nodes, highlighting significant spatial heterogeneity overall. In addition, in Figure 8a,b, most nodes appear as dark red, with spreaders and immune individuals dominating most of the space. The overall distribution trend is contrary to the ignorant individuals.

However, it is widely acknowledged that there exist various types of social networking platforms, and most internet users are only familiar with a few well-known ones. This leads to rumor spreaders being more inclined to enter these popular platforms to spread information. Nevertheless, the fixed probability of connecting edges in an ER random network fails to account for the optimal node connections. We will address this issue by leveraging the characteristics of BA scale-free networks.

In Figure 8d–f, the density distribution of S,I,R is depicted, and the parameter selection is consistent with that of the ER network. Comparing Figure 8d–f longitudinally with Figure 8a–c, there is a significant reduction in the number of nodes with different colors and a decrease in spatial heterogeneity. Given that nodes in the BA scale-free network tend to connect more frequently with highly connected nodes, relevant authorities can implement rumor-refuting policies for individuals located in key dark blue nodes during rumor control, thereby effectively controlling rumor propagation throughout the entire network and demonstrating high efficiency.

**Figure 9:**
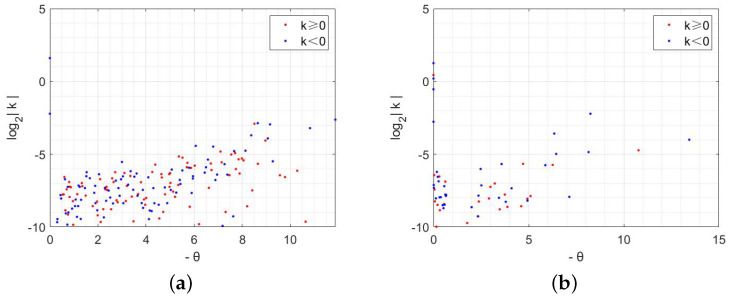
The distribution of coefficients after Fourier expansion of *S* in ER and BA networks.

Next, we proceed to verify the existence of the Turing pattern in Figure 8 by examining the spectral domain figure presented in Figure 9. Figure 9a,b illustrate the distribution of Fourier coefficients obtained from expanding with respect to *S* in an ER random network and BA scale-free network, respectively. It is evident that scatter points are more densely distributed within the eigenvalues ranging from −10 to 0, and a significantly higher density of scatter points is observed in Figure 9a compared to Figure 9b. In contrast to the concentrated Fourier coefficient distribution seen in the regular network shown in Figure 6, the overall distribution becomes more disordered due to accounting for randomness and is no longer confined solely along a reduced curve. Consequently, this transition from regularity to irregularity within the network structure considerably complicates exploring the distribution of Fourier coefficients. As a result, studying the changes for specific individuals occurring at each node within space becomes even more challenging.

### 5.2. The Case of Heterogeneous Networks

The flow of individuals in a homogeneous network is interconnected, but for effective rumor control, it is essential to limit the direction of individual flows. By restricting the entry and exit for different types of individuals in specific node connections, utilizing heterogeneous networks for analysis becomes more suitable for real-world scenarios, enabling efficient control.

**Figure 10:**
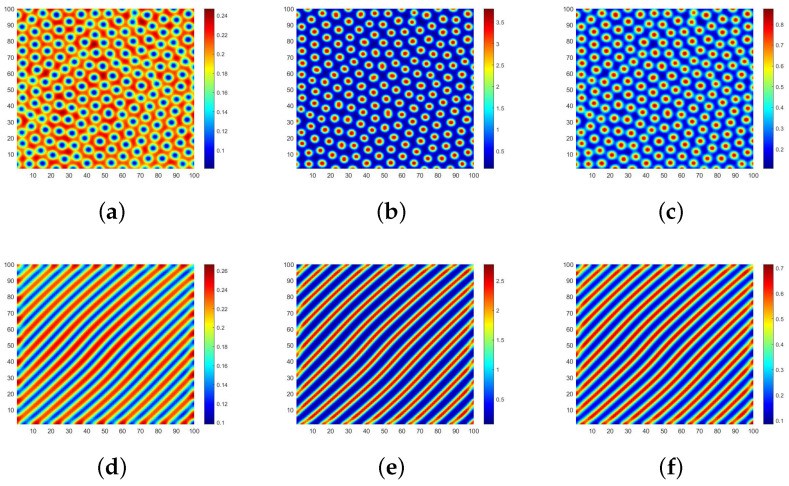
Turing patterns in QL–QL–OL and QL–HL–OL three-layer network structures.

In Figure 3, we investigate the case where the three-layer network structure is homogeneous. Subsequently, we break this assumption. Regarding parameter value selection, we maintain consistency with Figure 3a. Though the multitude of network combinations, the flow of the spreader *I* is the focus of attention in the process of rumor transmission, and it is also the direct source of relevant policies proposed by the power department. Based on these criteria, we set the flow environment of *S* and *R* with QL and OL network structures, respectively. Figure 10 illustrates scenarios where the flow environment of *I* corresponds to QL and HL network structures, respectively.

The result of the QL–QL–OL network structure is illustrated in Figure 10a–c, which represents the Turing patterns with respect to S,I and *R*, respectively. In comparison with Figure 3a–c, there is no significant change in the overall spatial pattern. Ignorant individuals *S* continue to be evenly distributed throughout the space, while spreaders *I* and immune individuals *R* are clustered together as spots. This implies that when the flow environment corresponding to *R* is modified independently and the average degree of the network increases, it does not have a substantial impact on the spatial distribution for different groups of populations. Figure 10d–f corresponds to the patterns of S,I,R for the scenario of the QL–HL–OL network structure. Notably, there are evident changes in the spatial distribution of all types of individuals, primarily forming striped patterns, with spreader and immune individuals exhibiting large-scale aggregation. By comparing Figure 10b,e, it becomes apparent that an increase in connecting edges between network nodes significantly amplifies the risk associated with rumor propagation. At this stage, the rumor spread enters a late state where conventional measures prove ineffective, thus necessitating stronger interventions to halt its spread.

**Figure 11:**
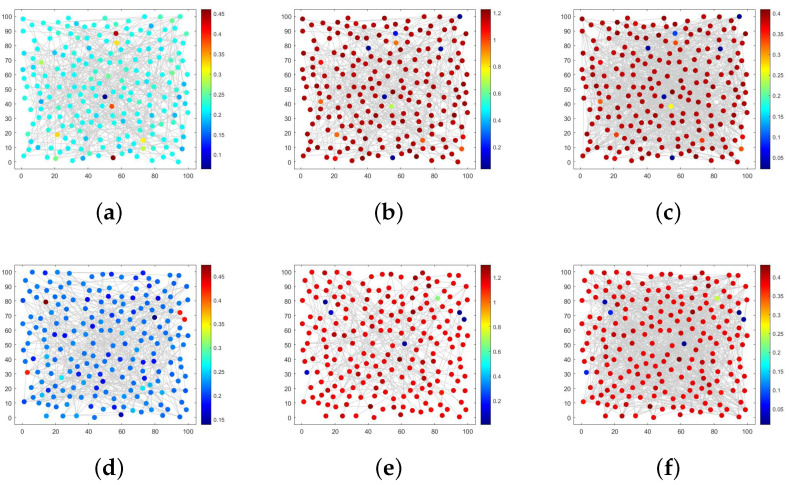
Turing patterns in ER–ER–ER2 and ER–BA–ER2 three-layer network structure.

Figure 11 illustrates the simulation outcomes of different irregular networks in a three-layer network. Parameters consistent with Figure 3a are still selected, assuming that the ER2 network has an edge connection probability of 0.04. Figure 11a–c display the Turing patterns corresponding to the ER–ER–ER2 network structure with respect to S,I and *R*, respectively. By increasing the edge connection probability, we observe a significant enrichment in node colors and strong spatial heterogeneity compared to the ER–ER–ER network structure shown in Figure 8. Due to real characteristics of the BA network, we replace the ER network corresponding to *I* with a BA network to obtain Figure 11d–f. Upon examining the longitudinal contrast, we can observe that there is a noticeable reduction in differently colored nodes.

When the network structure corresponding to *S* and *R* remains unchanged, altering the network structure associated with *I* will significantly impact the spatial distribution of individuals. This undoubtedly serves as a reminder to relevant departments that controlling the transmission channels of spreaders is crucial in the process of rumor control. Furthermore, it is essential to focus on spreaders in key nodes to enhance rumor control efficiency across the entire network. Compared to modifying the three-layer network structure for rumor control purposes, similar effects can be achieved by targeting specific layers, thereby greatly reducing the difficulty of rumor control.

## 6. Model Rationality Test

Throughout the global health crisis, digital platforms emerged as the primary engines for news circulation. Specifically, extracting emotional insights from Twitter became an essential tool for refining public health strategies and institutional communication. By leveraging quantitative frameworks, researchers can simulate data diffusion, measure policy efficacy, and forecast how message propagation might evolve. Guided by these objectives, we utilize System (2) as our core analytical framework, ensuring its empirical validity through focused case studies.

We curated a specialized dataset consisting of Indonesian Twitter discourse related to the COVID-19 outbreak, spanning a 54-day observation window from May through July 2020 (https://www.kaggle.com/datasets/dionisiusdh/covid19-indonesian-twitter-sentiment accessed on 7 February 2026). Adhering to the preprocessing protocols established in [18], our preliminary phase involved refining the raw text for analysis. To quantify the growth of the user base participating in information sharing, we computed the aggregate trajectory of tweet volumes over the study period. Furthermore, to neutralize potential biases arising from disparate population scales, we utilized the relative frequency of these participants as our primary calibration metric, which is formally identified as variable *N*. Consequently, the following System (17) derived from System (2) is constructed.

(17) $$\left\{\begin{array}{cc} \frac{\partial S(t,x)}{\partial t}=d_{1}\Delta S+rS\left(t,x\right)\left(1-\frac{S(t,x)}{K}\right)-\beta S\left(t,x\right)I^{2}\left(t,x\right)-\mu_{1}S\left(t,x\right), & t>0,\;x\in \Omega ,\\ \frac{\partial I(t,x)}{\partial t}=d_{2}\Delta I+\beta S\left(t,x\right)I^{2}\left(t,x\right)-\mu_{2}I\left(t,x\right)-\alpha I\left(t,x\right), & t>0,\;x\in \Omega ,\\ \frac{\partial R(t,x)}{\partial t}=d_{3}\Delta R+\alpha I\left(t,x\right)-\mu_{3}R\left(t,x\right), & t>0,\;x\in \Omega ,\\ \frac{\partial N(t,x)}{\partial t}=\beta S\left(t,x\right)I^{2}\left(t,x\right), & t>0,\;x\in \Omega ,\\ \frac{\partial S(t,x)}{\partial v}=\frac{\partial I(t,x)}{\partial v}=\frac{\partial R(t,x)}{\partial v}=\frac{\partial N(t,x)}{\partial v}=0, & t>0,\;x\in \partial \Omega ,\\ S(0,x)>0,\;I(0,x)\geq 0,\;R(0,x)\geq 0,\;N(0,x)\geq 0, & x\in \Omega . \end{array}\right.$$

**Figure 12:**
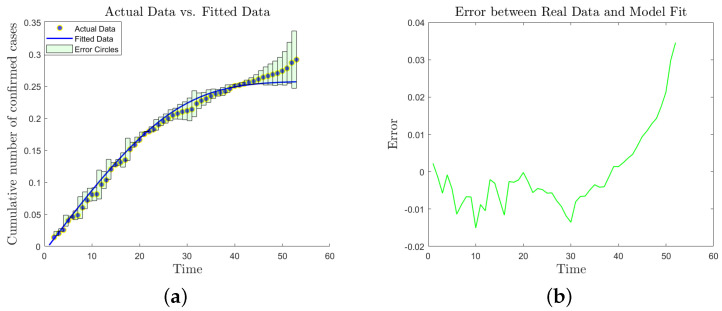
Model fitting based on real data from tweets.

We initialized the system states at S(x,0)=1, I(x,0)=0.1, R(x,0)=0.1, and N(x,0)=0.002, subsequently determining the optimal coefficients via a hybrid optimization strategy involving random walk heuristics and the least squares technique. The resulting parameter set (r=4.2125, $\mu_{1}=0.0045$, $\mu_{2}=0.0654$, α=0.0369, $\mu_{3}=0.5481$, K=0.9938 and β=0.9996) facilitates a high-fidelity representation of the data, as evidenced in Figure 12a,b. Quantitative assessment reveals that the divergence between our computational predictions and historical records remains largely below the 0.01 threshold, with a maximum deviation of 0.04. These minimal error margins demonstrate the superior accuracy and reliability of the current model.

**Figure 13:**
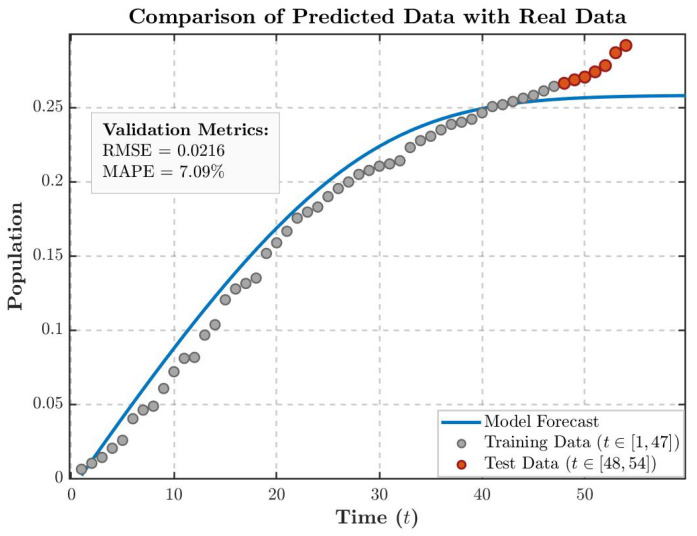
Comparison of predicted data with real data.

As illustrated by the comparison between the model forecast (solid blue line) and the training data (gray scatter points, t∈[1,47]) in Figure 13, the model successfully captures the dynamic characteristic of an approximate S-shaped growth in the cumulative infected population (*E*) during the early and intermediate phases. The overall fitting error is maintained within a reasonable range, with a Root Mean Square Error (RMSE) of 0.0216 and a Mean Absolute Percentage Error (MAPE) of 7.09%, which confirms the validity of the model’s parameter calibration.

**Figure 14:**
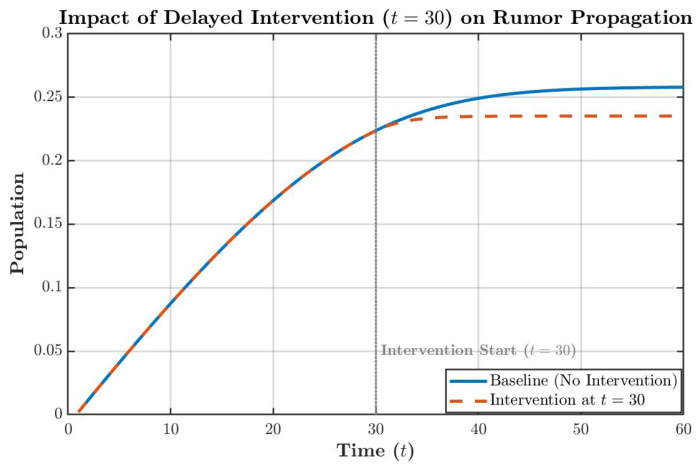
Impact of delayed intervention on rumor propagation.

While the model exhibits high fidelity during the initial phase, the divergence observed in the testing period (Figure 13) suggests a potential ’boundary-breaking’ escalation of the rumor. To evaluate the efficacy of administrative countermeasures in curbing such risks, an intervention scenario was simulated. By augmenting the recovery rate parameter α at t=30, we assessed the impact of proactive debunking on rumor suppression, as illustrated in Figure 14. As illustrated in Figure 14, implementing dynamic interventions before the rumor enters its exponential outbreak phase can effectively compel the spreading system to reach a stable equilibrium prematurely, thereby significantly curtailing the ultimate cumulative scale of the rumor’s impact.

## 7. Conclusions

Aiming to address the increasingly prominent and challenging issue of Internet rumor spread, this paper proposes a three-layer network structure model. Firstly, we reconstruct the diffusion term by utilizing the Laplacian matrix to replace the flow direction among three different types of individuals during rumor propagation. In the theoretical part, we investigate Turing instability conditions in homogeneous networks. Additionally, we employ spectral theory to resolve the problem of perturbations in heterogeneous networks that can not be expanded within the feature space of a Laplacian matrix and give the necessary conditions for Turing instability.

The research findings indicate that in a regular network, if the three-layer structure is homogeneous, the shape of the Turing pattern remains unchanged as the degree of each node increases. However, the spatial distribution pattern of the crowd has changed, which poses challenges for rumor control efforts. Conversely, if the three-layer network is heterogeneous, altering the network average degree corresponding to spreaders leads to changes in the shape of Turing patterns and significantly amplifies the risk associated with rumor propagation.

In order to restore the characteristics of the actual network, when analyzing an irregular network, if the three-layer network is homogeneous, it significantly reduces the spatial heterogeneity of the BA network compared to the ER network. This provides a theoretical basis for management departments to seize key nodes and effectively enhance rumor control efficiency. If the three-layer network is heterogeneous, it can be found that we can also achieve the effect of changing the entire three-layer network structure on rumor governance by changing the key layer, which brings positive implications for reducing difficulty in rumor control. We utilize a specialized data of Indonesian COVID-19 tweets to test the practical utility of our system. The resulting alignment between computational forecasts and historical observations confirms that the model accurately mirrors real-world rumor propagation with superior precision.

In this paper, we described the actual distribution and propagation states of various individuals through Turing patterns for different network structures. However, with the development of networks and intelligent technologies, the method of information dissemination and diffusion has changed. Social networks exhibit multi-agent high-order interactions; that is, multiple individuals participate in group communication and dissemination. For this more realistic network structure, we can attempt to achieve characterization and analysis through Turing patterns. In addition, we will collect more abundant real social dissemination data for empirical analysis, striving to enhance the application value of the theory.

## Figures and Tables

**Table 1:** Parameter interpretation.

Parameter	Interpretation
*r*	The natural growth rate of individuals susceptible to rumors.
*K*	The maximum carrying capacity of space.
$\mu_{1},\mu_{2},\mu_{3}$	The replacement rate of individuals for S,I,R, respectively.
β	The rate at which rumors spread.
α	Recovery rate of spread individuals due to information communication.

## Data Availability

The data for the reasonableness test of the model were obtained from the following website: https://www.kaggle.com/datasets/dionisiusdh/covid19-indonesian-twitter-sentiment accessed on 7 February 2026).
